# Modeling the Current and Future Distribution of *Indianthus virgatus* (Roxb.) Suksathan & Borchs.: A Monotypic Plant Endemic to the Western Ghats‐Sri Lanka Biodiversity Hotspot

**DOI:** 10.1002/ece3.70489

**Published:** 2024-10-26

**Authors:** Shreekara Bhat Vishnu, Vivek Pandi, Indrakheela Madola, Bhathiya Gopallawa, Gija Anna Abraham, Rajendiran Gayathri, Deepthi Yakandawala, Annamalai Muthusamy

**Affiliations:** ^1^ Manipal Centre for Natural Sciences Manipal Academy of Higher Education Manipal India; ^2^ Department of Horticulture and Landscape Gardening, Faculty of Agriculture and Plantation Management Wayamba University of Sri Lanka Kuliyapitiya Sri Lanka; ^3^ Department of Botany University of Peradeniya Kandy Sri Lanka; ^4^ Zoological Survey of India Southern Regional Centre Chennai India; ^5^ Department of Plant Sciences, Manipal School of Life Sciences Manipal Academy of Higher Education Manipal India

**Keywords:** climate change, conservation, IUCN, MaxEnt modeling, species distribution modeling, Wallace ecological modeling

## Abstract

Species distribution modeling (SDM) is an essential tool in ecology and conservation for predicting species distributions based on species presence/absence data and environmental variables. The present study aimed to understand the distribution pattern and habitat suitability of *Indianthus virgatus* under current and future climate change scenarios (2050 and 2070) using *MaxEnt (3.4.4)* and *Wallace Ecological Modeling (v2.1.2)* tools. The study also intended to identify key environmental predictors of *I. virgatus'* distribution. Species occurrence data were collected from various sources, including herbarium (online and physical), field surveys, and online databases, yielding 105 unique locations in the Western Ghats (WG) of India and Sri Lanka. We used 19 bioclimatic variables and elevation data sourced from WorldClim for modeling. The *MaxEnt* and *Wallace* models showed excellent performance in predicting the distribution of *I. virgatus*, with area under the curve values of 0.958 (± 0.002) and 0.93, respectively. In *MaxEnt* modeling, Temperature Seasonality (bio4) was the most significant environmental parameter, followed by the Precipitation of the Coldest Quarter (bio19). In contrast, the Annual Mean Temperature (bio1), Temperature Seasonality (bio4), and Annual Precipitation (bio12) were among the key contributors in *Wallace EcoMod*. Both the models predicted relatively lesser areas in the species' distribution range as highly suitable habitats (HSH) in India and Sri Lanka. We found divergent trends in predicting *I. virgatus* distributions using *MaxEnt* and *Wallace EcoMod*, particularly for future projections. Nevertheless, both models predicted significant habitat loss under future climate change scenarios, especially under RCP85, with varying degrees of suitability across India and Sri Lanka. Overall, our findings on expected habitat loss under future climate change scenarios highlight the importance of conserving *I. virgatus*, which has already been declared critically endangered (CR) in Sri Lanka.

## Graphical abstract

This article projected the distribution of *Indianthus virgatus*, a monotypic plant endemic to the Western Ghats–Sri Lanka biodiversity hotspot. The article also highlights the role of environmental parameters in determining the current and future distribution projections using *MaxEnt* and *Wallace EcoMod*.

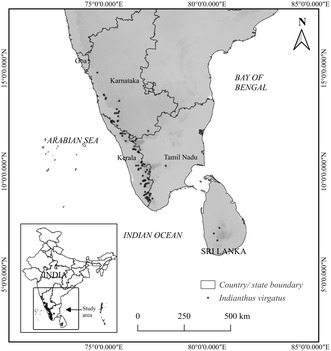

## 1 Introduction

The role of climate change is inevitable in altering ecological landscapes, posing potential challenges to biodiversity worldwide (Guisan et al. 2013; McCune 2016; Mathur and Mathur 2023). The impact of climate change demands species adapt various mechanisms, including migration, shifts in phenological cycles, and the development of novel physiological traits (Behera, Behera, and Sharma 2019; Mathur and Mathur 2023) to support their fitness and survival. According to the Millennium Ecosystem Assessment (2005), climate change is poised to emerge as one of the primary drivers of biodiversity decline by the end of this century. The repercussions of climate change profoundly affect various plant and animal species, posing significant threats to their survival and ecological equilibrium (Sousa‐Silva et al. 2014; Ray et al. 2019).

Under the current climate change scenario, understanding the intricate relationship between climate dynamics and species distributions and the potential consequences of future climatic shifts on range adjustments has become critical. Over the last three decades, there has been an enormous effort to enhance and utilize species distribution models (SDMs) to elucidate these phenomena (Radosavljevic and Anderson 2014; Araújo et al. 2019; Santini et al. 2021). SDM examines the ecological/environmental demands and limits of species throughout space and time, utilizing a variety of techniques based on statistical algorithms (Valavi et al. 2022, 2023). In climate change forecasting studies that use SDMs, the models are trained on current information to predict the likelihood of species occurrence under present and future environmental conditions. Binarizing model predictions is a typical approach for determining whether a species' distribution will shift, contract, or expand in response to climate change (Liu et al. 2013; Guisan et al. 2013; Newbold 2018; Santini et al. 2021).

**PLATE 1:**
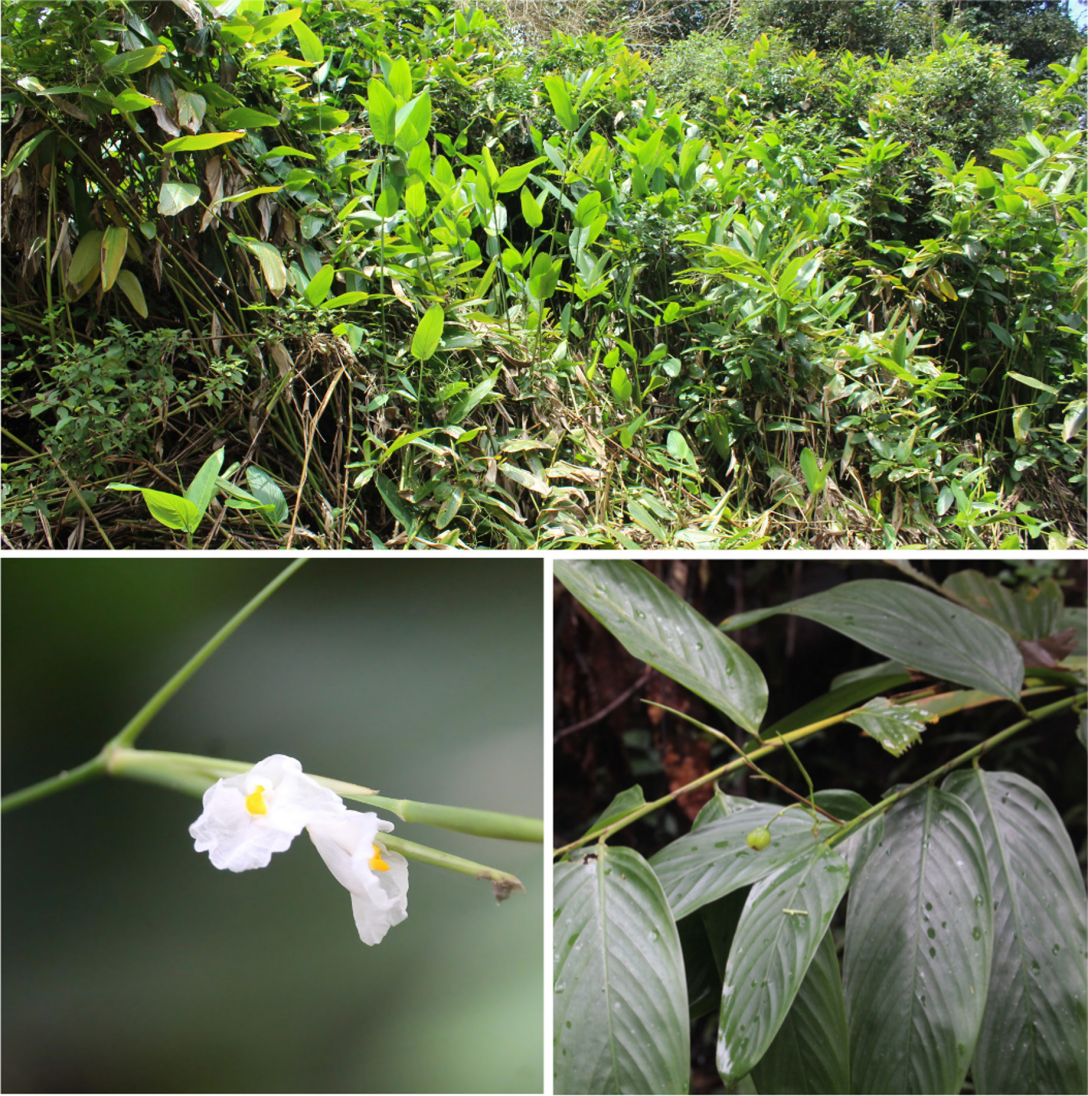
*Indianthus virgatus* (Roxb.) Suksathan & Borchs. in its natural habitat in the Western Ghats.

SDMs are primarily performed for species of ecological/economic and conservation significance (e.g., Elith et al. 2006, 2011; Syfert et al. 2014; Ali et al. 2023; Mirhashemi et al. 2023; Qazi et al. 2023). Within the family Marantaceae, the monotypic genus *Indianthus* includes the species, *Indianthus virgatus* (Roxb.) Suksathan and Borchs., endemic to the WGs of peninsular India and Sri Lanka (Suksathan et al. 2009; Narasimhan and Irwin 2021; Govaerts 2011). *I. virgatus* represents a relict species confined to the wet evergreen forests, moist slopy hills, and Myristica swamps of the WGs and Sri Lanka (Plate 1). *I. virgatus* has long been used medicinally (Sangeetha and Rajamani 2019) and commercially by the local communities. Its specific niche requirements, endemism, and unregulated use highlight the potential risk for this monotypic taxon. According to the National Red List (2020) of Sri Lanka, *I. virgatus* is critically endangered (CR) in its native range in Sri Lanka. The IUCN status of *I. virgatus* in India has yet to be assessed. Therefore, it is imperative to assess its current distribution and future projections using climate change models. Using the precise geographical details collected from fieldwork and literature sources, we perform SDM for *I. virgatus*, asking the following questions:

1. What is the current distribution pattern of *I. virgatus* in India and Sri Lanka?
2. What are the primary environmental variables that contribute significantly to the distribution of *Indianthus*?
3. What is the projected habitat suitability of *I. virgatus* under changing climatic conditions in 2050 and 2070, using *MaxEnt* and *Wallace* tools with CCSM4 and the RCP26, RCP45, RCP60, and RCP85 global climate change scenarios?
4. How does climate change influence the habitat range expansion/contraction of *I. virgatus* over time?

## 2 Materials and Methods

### 2.1 Occurrence Data

**FIGURE 1:**
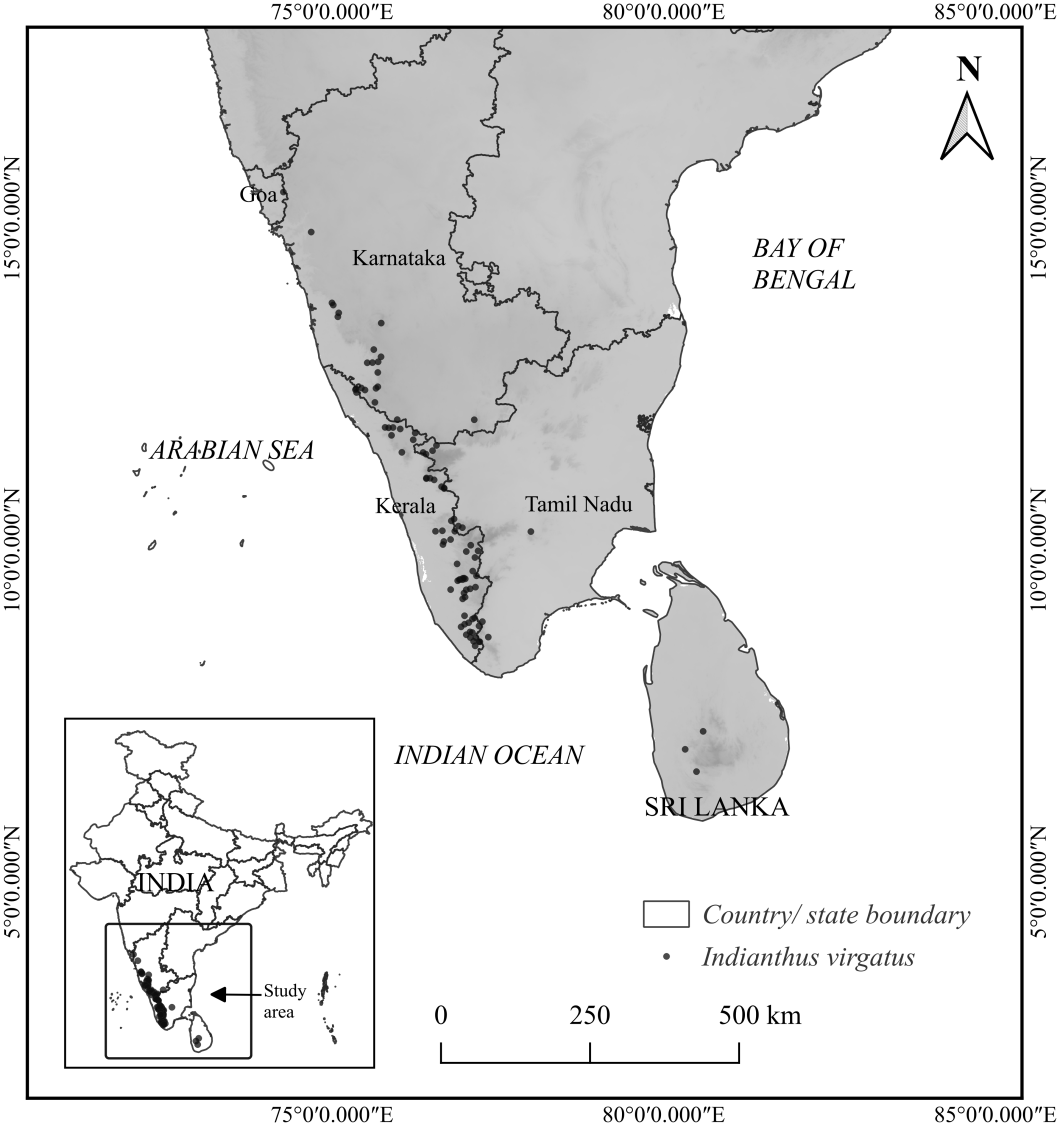
Occurrence records of *Indianthus virgatus* in India and Sri Lanka.

Species presence data for *Indianthus virgatus* were collected from multiple physical repositories such as herbarium records and flora books (Narasimhan and Irwin 2021; Bhat 2014; Sankara Rao et al. 2016). Herbarium data were obtained from the Central National Herbaria, Botanical Survey of India (BSI) Kolkata, BSI Coimbatore, BSI Pune, French Institute of Pondicherry herbarium (HIFP), Calicut University herbarium (CALI), Jawaharlal Nehru Tropical Botanic Garden and Research Institute herbarium (JNTB), and Kerala Forest Research Institute herbarium (KFRI). A few other online herbarium databases and digital repositories, including the Global Biodiversity Information Facility GBIF (https://www.gbif.org/citation‐guidelines), Indian Biodiversity Portal, Flowers of India (https://www.flowersofindia.net/catalog/slides/White%20Indianthus.html), eFlora of India (https://efloraofindia.com/2011/11/03/schumannianthus‐virgatus/), and Digital Flora of Eastern Ghats, IISc JCB (http://easternghats.ces.iisc.ernet.in/plants.php?name=Schumannianthus) were also accessed for occurrence data. In addition, extensive fieldwork was carried out between March 2023 and April 2024 to verify and validate the occurrence data across the WGs and Sri Lanka. GPS coordinates were recorded in the field with a Garmin ETREX 32X (Garmin (Asia) Corporation, Taiwan). The distribution records (latitude and longitude) were subsequently compiled in an Excel spreadsheet and converted to CSV format for further analysis. Duplicate records and records without precise locations were removed from the dataset. Overall, the literature survey a fieldwork generated 105 unique, validated locations within the species' distribution range (Figure 1; Supporting Information S1). A total of 60 locations were used after applying the spatial thinning at a distance of 10‐km radius.

### 2.2 Environmental Variables for SDM Using *MaxEnt* (Current and Future)

The environmental variables included 19 bioclimatic parameters and elevation data (Table 1). Current climate data (1970 to 2000) were collected from the WorldClim database (Hijmans et al. 2005; http://www.worldclim.org). Elevation data was obtained from the digital elevation SRTM database version 4.4 (Hijmans et al. 2005). A resolution of 2.5 arc seconds was used to maintain projection precision while optimizing the environmental factors required for species distribution modeling. The study covered the current period (averages from 1970 to 2000), the 2050s (averages from 2041 to 2060), and the 2070s (averages from 2061 to 2080). The climate variables were derived from the Community Climate System Model version 4 (CCSM4). Future climatic variables were obtained using the Representative Concentration Pathway (RCP) scenarios, notably the RCP26, RCP45, RCP60, and RCP85, following Liu et al. (2021). To enhance the precision of the *MaxEnt* model and address potential spatial collinearity among environmental variables, we implemented several steps. Initially, we imported the distribution data for *I. virgatus* along with all associated environmental variables (Table 1) into *MaxEnt*. We divided the distribution data, allocating 25% for testing and 75% for training purposes. Using default settings, we executed the calculations five times employing the bootstrapping method. Additionally, the number of iterations and background points was configured to 10,000. In addition, we used the jackknife test to determine the percent impact of each environmental variable, following Phillips, Anderson, and Schapire (2006). Environmental variables with low predictive value were then excluded from the modeling process.

**TABLE 1:** Environmental variables for the prediction of the distribution (current and future) of *Indianthus virgatus*.

Type	Variables	Description
Bioclimatic variables	Bio1	Annual mean temperature (°C)
	Bio2	Mean diurnal range [mean of monthly (max temp − min temp)] (°C)
	Bio3	Isothermality (Bio2/Bio7) (×100)
	Bio4	Temperature seasonality (SD × 100; Coefficient of variation) (°C)
	Bio5	Max temperature of warmest month (°C)
	Bio6	Min temperature of coldest month (°C)
	Bio7	Temperature annual range (Bio5‐Bio6) (°C)
	Bio8	Mean temperature of wettest quarter (°C)
	Bio9	Mean temperature of driest quarter (°C)
	Bio10	Mean temperature of warmest quarter (°C)
	Bio11	Mean temperature of coldest quarter (°C)
	Bio12	Annual precipitation (mm)
	Bio13	Precipitation of wettest month (mm)
	Bio14	Precipitation of driest month (mm)
	Bio15	Precipitation seasonality (coefficient of variation)
	Bio16	Precipitation of wettest quarter (mm)
	Bio17	Precipitation of driest quarter (mm)
	Bio18	Precipitation of warmest quarter (mm)
	Bio19	Precipitation of coldest quarter (mm)
Topography	Bio20	Elevation

### 2.3 Species Distribution Modeling Using *Wallace*

For the *Wallace* modeling, *Wallace* was installed from CRAN in RStudio 4.3.2. *Wallace* was run using the detailed methodology outlined by Kass et al. (2018). The default browser opened *Wallace 2.1.1*, where the occurrence data was uploaded in CSV format. Similar to *MaxEnt*, 19 bioclimatic factors at 2.5 arc‐minute spatial resolution were used for the prediction. The dataset was then partitioned, with 25% labeled for test data and the remaining 75% used as training data. Using the occurrence records, 10,000 predicted points were constructed, with spatial thinning used to eliminate duplicate entries within a 10 km radius. Using *Wallace*'s *MaxEnt* module, the regularization multiplier was chosen between 1 and 2.5. Various feature classes, such as L, LQ, H, LQH, and LQHP, were used to improve the model's predictive ability. Map predictions, *MaxEnt* assessment charts, and response curves were then generated and saved for later evaluation. AUC (area under the curve) and delta Akaike information criterion (AICc) values were used to compare model performance against random prediction. To assess the complexity and flexibility of our species distribution model, we employed several evaluation metrics. These included the delta values from both the AIC and AICc, the difference between the training and testing AUC (AUC.DIFF), and the 10% training omission rate (OR10) as detailed by Vallecillo Rodriguez et al. (2016). The model was then moved to a new time frame and projected for RCP26, RCP45, RCP60, and RCP85 scenarios using the CCSM4 climate model, with a resolution of 2.5 arc minutes. The Receiver Operating Characteristic (ROC) and AUC were used to measure the model's quality, which is independent of the critical value in the model.

This evaluation approach assesses the model's correctness (Zhu, Zhang, and Huang 2017; Wan et al. 2023). AUC values range from 0 to 1, with values less than 0.7 indicating extremely poor prediction accuracy, values between 0.7 and 0.8 suggesting moderate performance, values between 0.8 and 0.9 indicating good performance, and values between 0.9 and 1.0 indicating excellent performance (Muscarella et al. 2014). Essentially, a higher AUC value suggests a more accurate model fit.

### 2.4 Partitioning Potential Area for Indianthus Using QGIS

We imported the ASCII files from *MaxEnt* into QGIS 3.32.1 and transformed them into grid data, which we then overlaid on the administrative zoning map for visual analysis. Using GIS's reclassification tool, we classified regions according to their suitability index for *I. virgatus*. Our classification system categorized areas into four groups based on the suitability index, P, as follows:

- Unsuitable habitat (0.0–0.20)
- Least suitable habitat (LSH) (0.21–0.40)
- Moderately suitable habitat (MSH) (0.41–0.70)
- Highly suitable habitat (HSH) (0.71–1.0)

To calculate the area in each suitability class, we used the raster layer unique values report tool from the QGIS toolbox. Since raster data is composed of pixels, the area covered by each class is derived by multiplying the number of pixels in that class by the area of a single pixel. The pixel area (in square kilometers) was determined based on the spatial resolution of the raster. After the computation was done for each area, we summarized the total area of suitability for *I. virgatus* (Guisan and Zimmermann 2000; Jenness 2004).

## 3 Results

### 3.1 Model Results—*MaxEnt* Modeling

**FIGURE 2:**
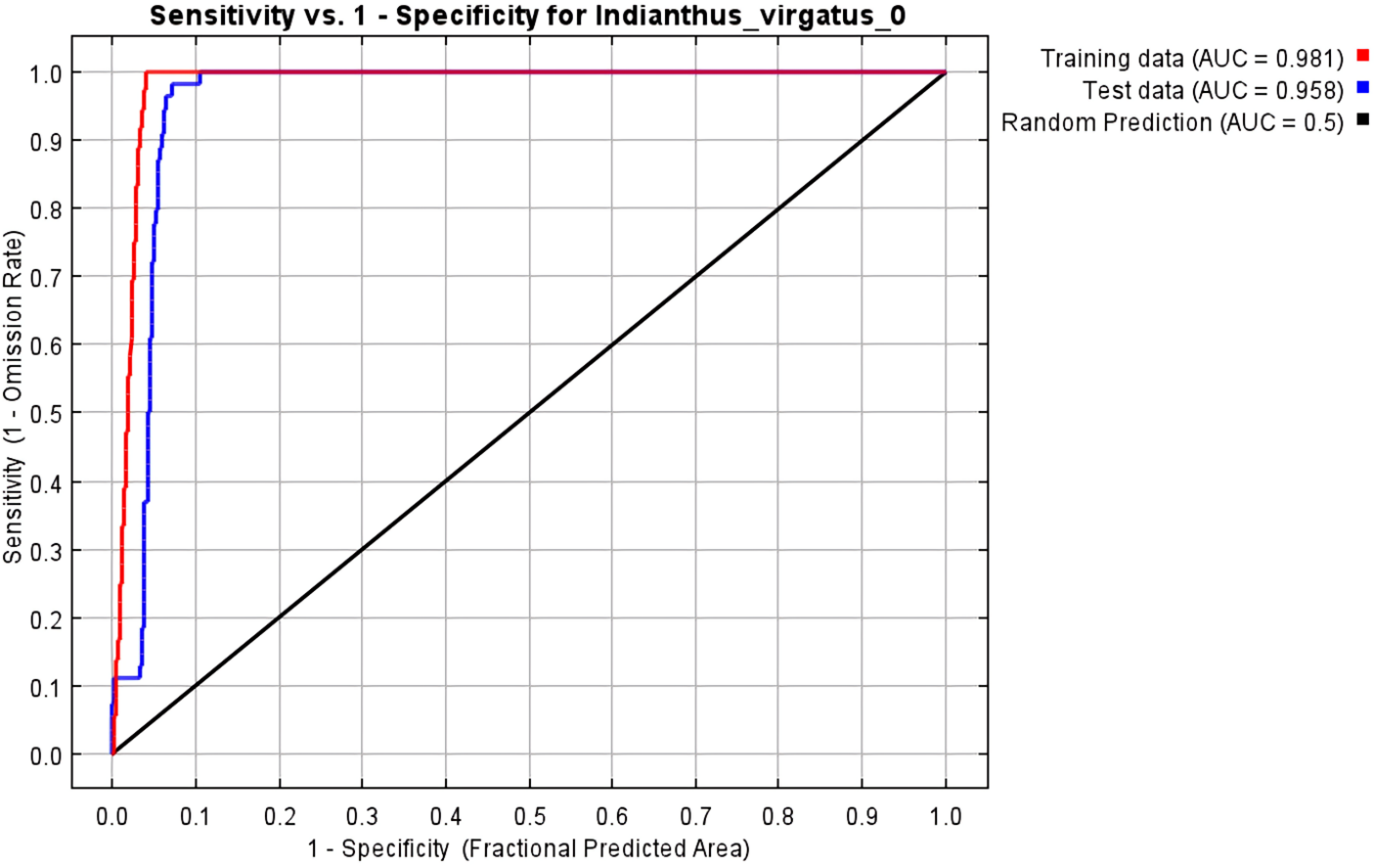
AUC values for the training and test data using the *MaxEnt* model.

**FIGURE 3:**
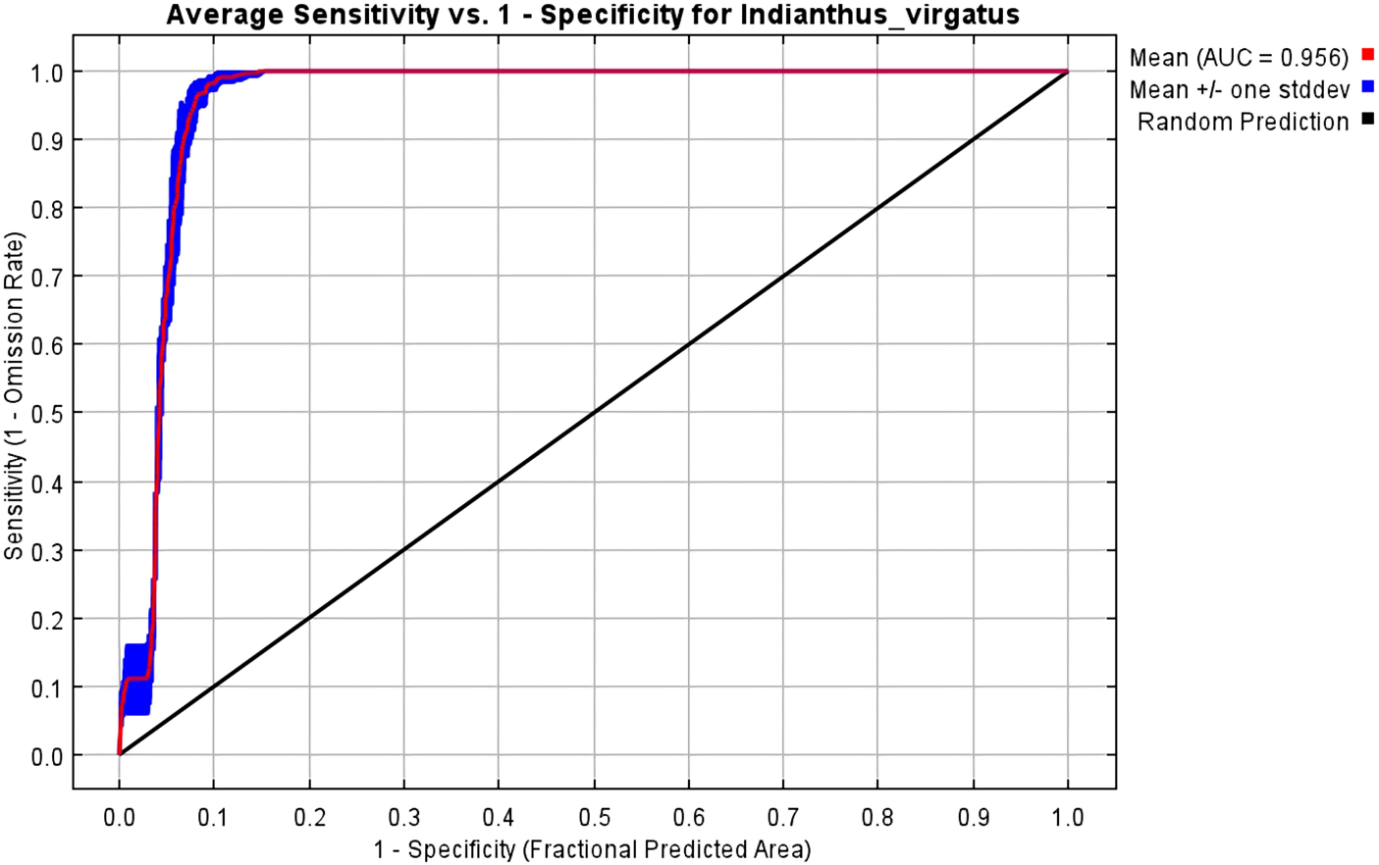
Mean AUC values for *I. virgatus*.

**FIGURE 4:**
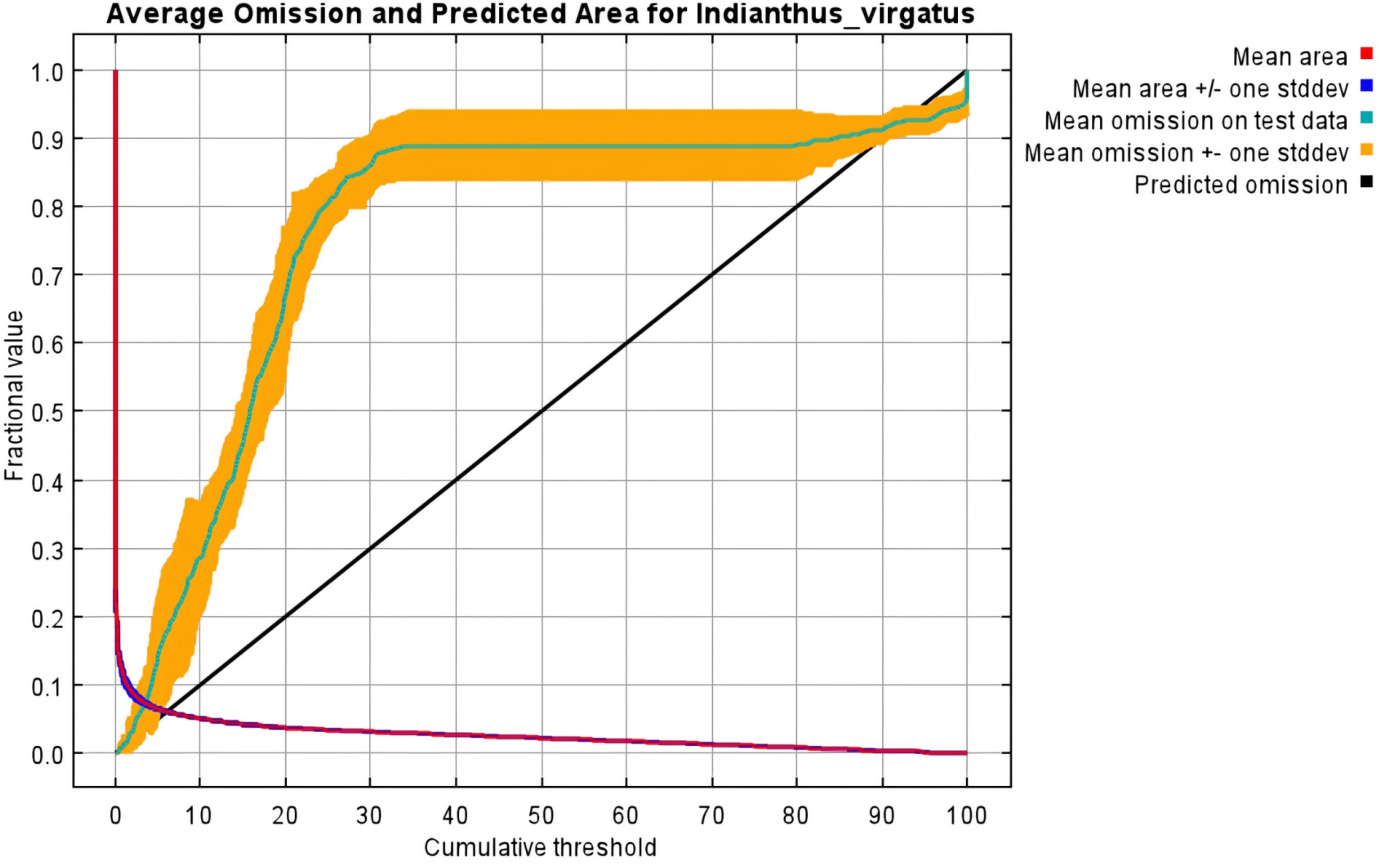
Omission value for the predicted area.

**FIGURE 5:**
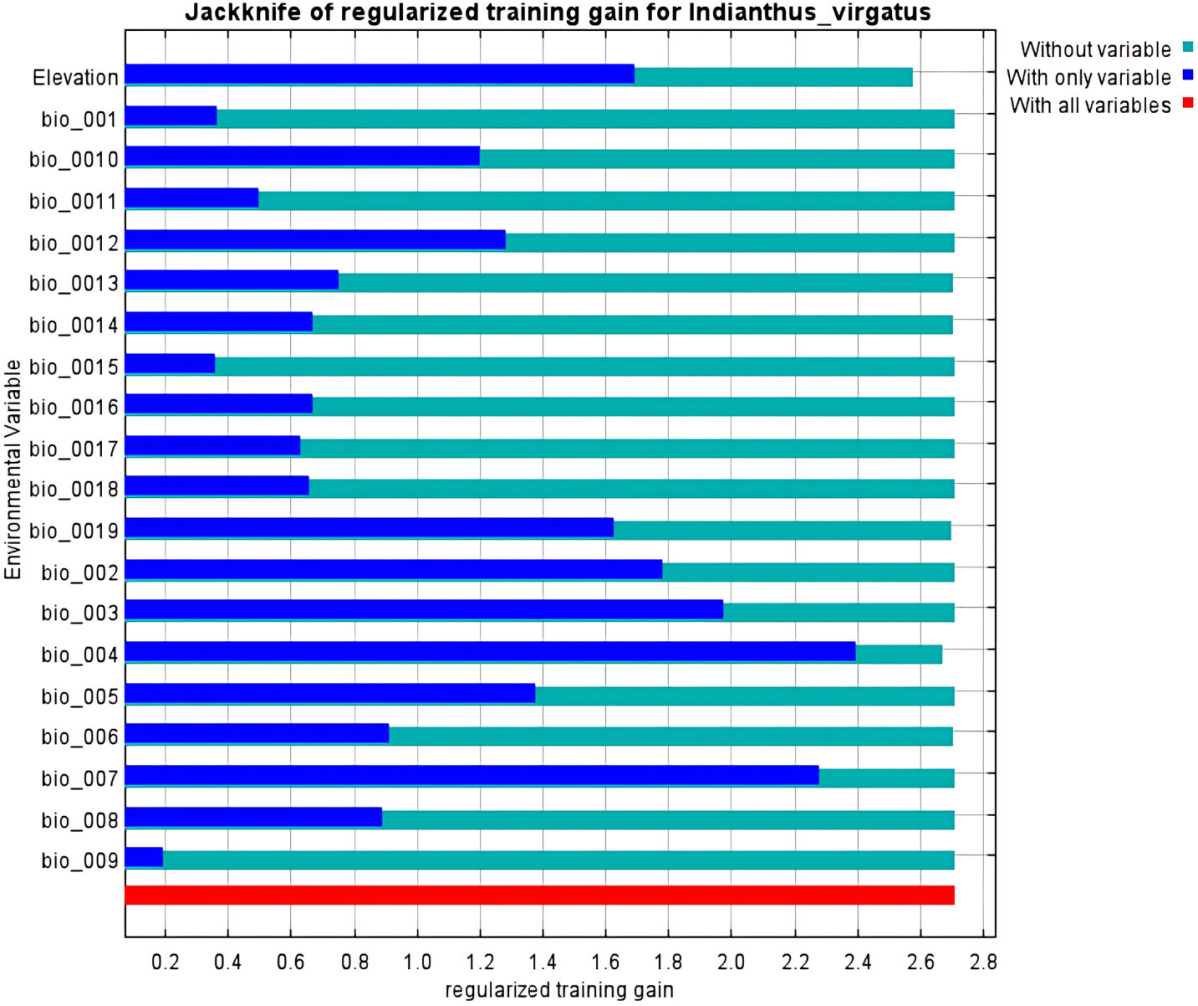
Relative prediction of environmental variables through the Jackknife test for *I. virgatus*.

**FIGURE 6:**
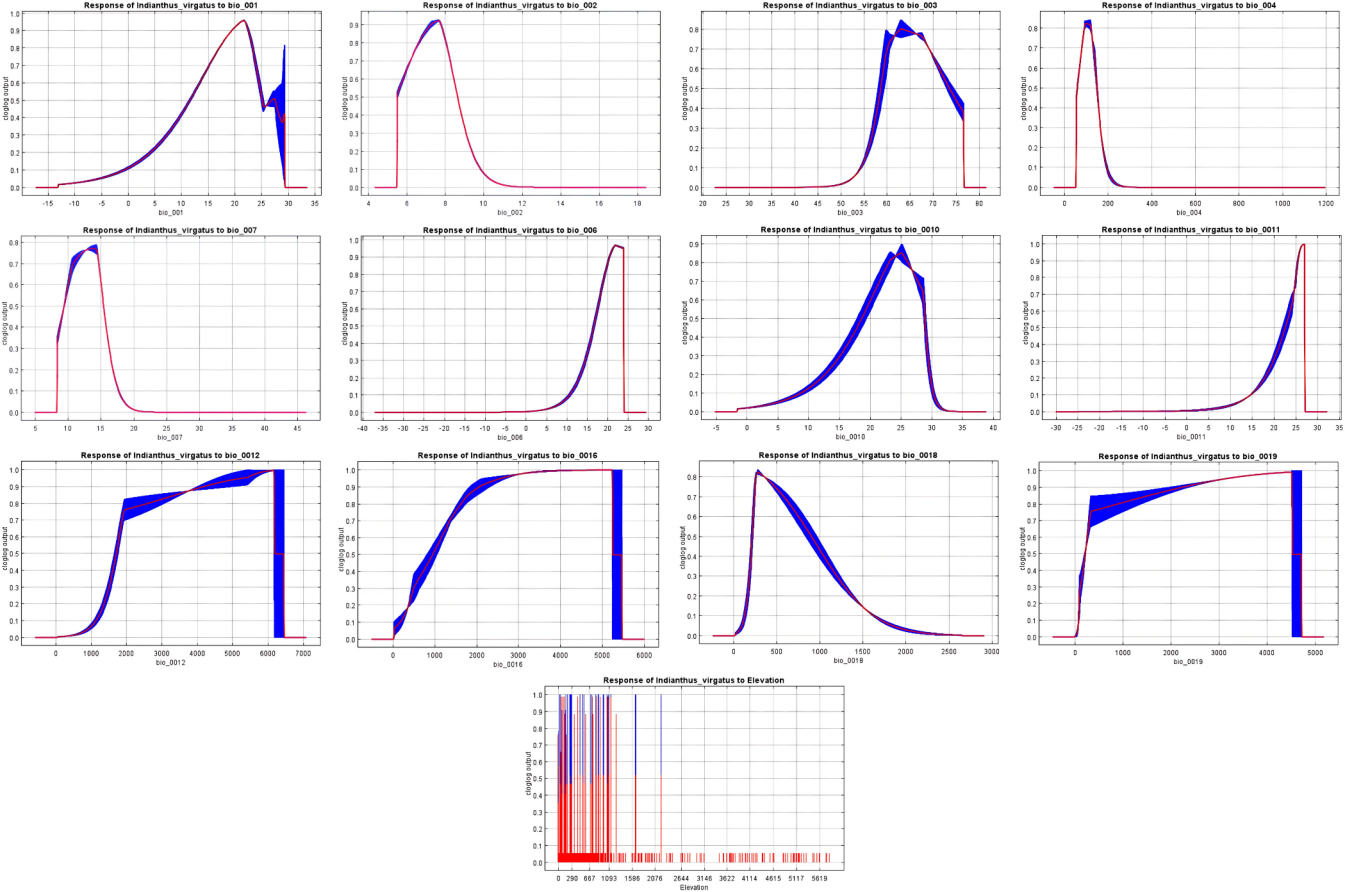
Threshold of environmental variables against *I. virgatus* distribution.

With an AUC value of 0.958 (± 0.002), the *MaxEnt* model applied in the present study demonstrated excellent performance compared to the random model with an AUC value of 0.5 (Figures 2 and 3). The test omission rate and predicted area against the cumulative threshold averaged across multiple runs. The test omission rate closely matches the predicted omission rate, indicating the model's accuracy in identifying true presences and absences (Figure 4). Based on the Jack‐knife test (Figure 5), Temperature Seasonality (bio4) was the most significant parameter in the model, followed by the Precipitation of the Coldest Quarter (bio19) (Table 2). Besides, Elevation (ELV), Isothermality (bio2/bio7), Annual Precipitation (bio12), and Temperature Annual Range (bio7) also contributed significantly to the model (Figure 6). These six parameters together contributed to 94.2% of the model's prediction of *I. virgatus*' distribution. The permutation importance of the top three predictors totaled 98.6% (Table 2).

**TABLE 2:** Percentage contribution of environmental variables for *I. virgatus* Maxent modeling predictions (current period, 1970–2000).

Code	Environmental variables	Percentage contribution (%)	Permutation importance (%)
BIO4	Temperature seasonality (standard deviation ×100)	44.9	94.9
BIO19	Precipitation of coldest quarter	29.8	0.7
ELV	Elevation	8	3
BIO3	Isothermality (BIO2/BIO7) (×100)	4.9	0
BIO12	Annual precipitation	3.6	0
BIO7	Temperature annual range (BIO5–BIO6)	3	0
BIO2	Mean diurnal range (mean of monthly (max temp − min temp))	1.7	0.4
BIO6	Min temperature of coldest month	1.3	0.2
BIO18	Precipitation of warmest quarter	1.2	0
BIO16	Precipitation of wettest quarter	0.6	0.2
BIO1	Annual mean temperature	0.5	0
BIO11	Mean temperature of coldest quarter	0.2	0
BIO10	Mean temperature of warmest quarter	0.1	0

### 3.2 Predicted Current Distribution of *I. virgatus*

**FIGURE 7:**
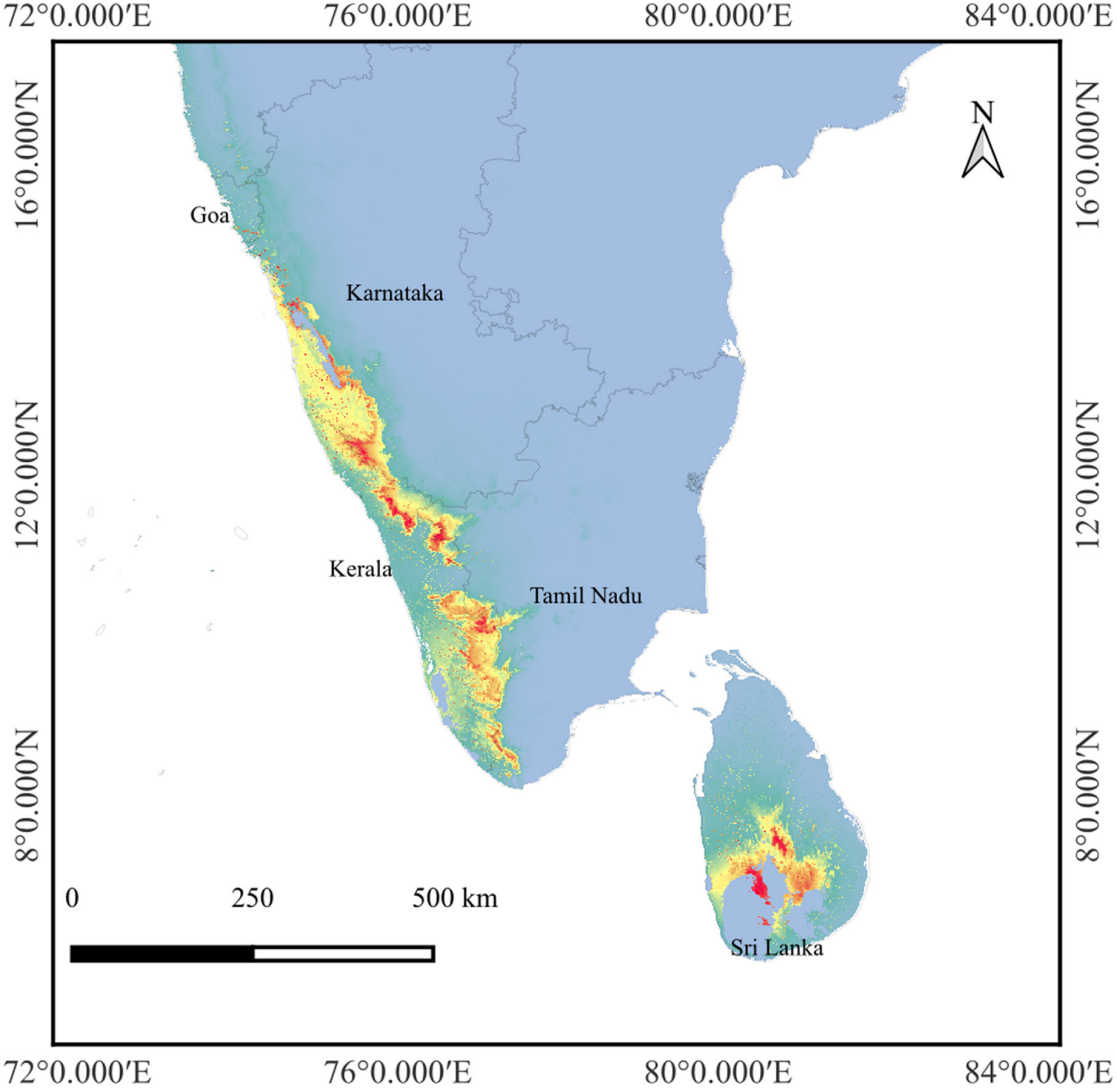
Present data (1950–2000) using *MaxEnt*.

The predicted HSHs for *I. virgatus* comprised just 12% (6707 km^2^) of the total area of species occurrence in India and 19% in Sri Lanka (Table 3; Figure 7). Further, the analysis using the reclassify tool in QGIS revealed that the MSHs of *I. virgatus* are primarily concentrated in the southern part of the Central Western Ghats (CWG) and throughout the Southern Western Ghats (SWG) in India, where the presence of *I. virgatus* is validated. Although negligible, the model also predicted the presence of the LSHs for *I. virgatus* in the Northern Western Ghats (NWG), where the family Marantaceae has not been reported so far. The Central Province comprised the highest habitat suitability for *I. virgatus* in Sri Lanka.

**TABLE 3:** *Maxent* modeling predicted area of suitability in India and Sri Lanka.

Province/state		RCP scenario	Highly suitable habitat (km^2^)	Moderately suitable habitat (km^2^)	Least suitable habitat (km^2^)
India	Current		6706.73	23,944.72	37,187.39
	2050	RCP26	7542.46	26,455.51	34,876.33
		RCP45	9097.2	23,326.25	45,883.46
		RCP60	5316.91	12,581.76	38,574.33
		RCP85	6112.5	12,010.8	55,954.55
	2070	RCP26	4967.08	16,458.69	35,291.83
		RCP45	9932.7	22,4308.52	42,650.98
		RCP60	9833.13	30,631.56	41,308.49
		RCP85	2333.01	24,494.7	45,525.5
Sri Lanka	Current		1440.335	2890.6	3135.45
	2050	RCP26	1680.55	2275.55	8200.44
		RCP45	1315.742	2250.237	2997.071
		RCP60	1263.39	2463.34	3317.85
		RCP85	1274.303	3094.973	2541.331
	2070	RCP26	1710.196	3853.825	6481.714
		RCP45	1655.183	2068.808	3376.7
		RCP60	711.512	3468.4	4141.13
		RCP85	634.84	3100.556	4442.345

### 3.3 Predicted Future Distribution of *I. virgatus* Distribution Using CCSM4 Under Different RCP Scenarios

**FIGURE 8:**
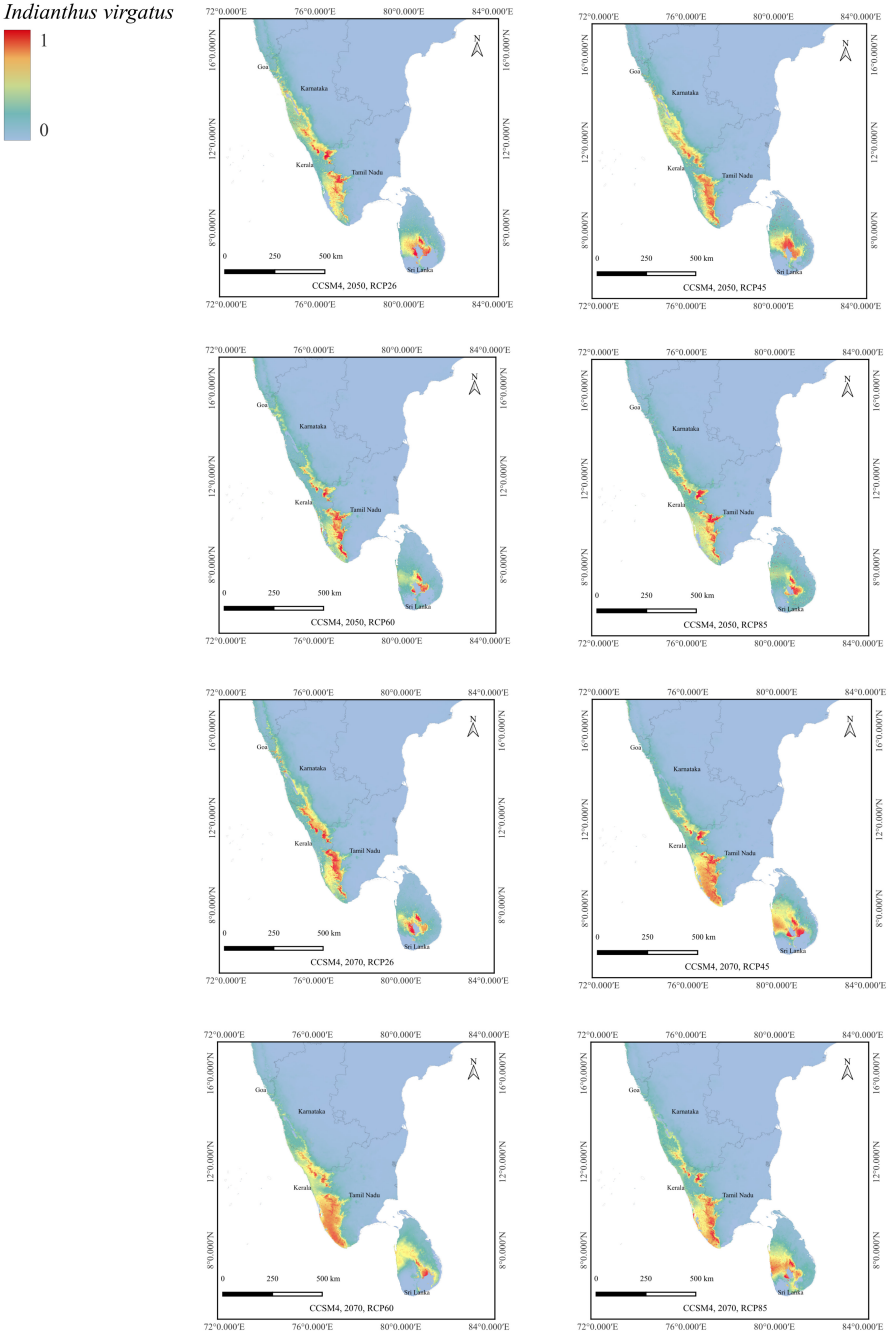
Future predictions for *I. virgatus* under CCSM4 2050 and 2070 RCP 26,45,60,85.

The projected potential suitable habitat for *I. virgatus* under future climate change scenarios of RCP 2.6, RCP 4.5, RCP 6.0, and RCP 8.5 for the years 2050 and 2070 revealed significant differences in suitable habitats between current distribution and those predicted for the 2050s and 2070s (Figure 8). The magnitude of changes also varied across different RCP scenarios. Under the RCP85 scenario, there was a more pronounced reduction in the suitable habitat compared to other scenarios in India and Sri Lanka. The predicted transition of the current extent of HSH habitat from 6706.73 km^2^ to 2333.01 km^2^ under the RCP85 scenario for 2070 indicated a significant loss (−65.19%) in the species distribution range in India (Table 3). Similarly, the model indicated a significant loss of HSH by −55% under RCP85 for 2070 in Sri Lanka. However, the model also predicted substantial expansion in the extent of HSHs for *I. virgatus* under RCP45 and RCP60 for 2070, predominantly in the SWGs. Also, under the RCP26 and RCP45 scenarios for 2050, a significant range expansion was predicted in India in the HSH category (12.46% and 35.89%, respectively) (Table 3).

### 3.4 Wallace Ecological Modeling—Model Results, Current and Future Predictions

**FIGURE 9:**
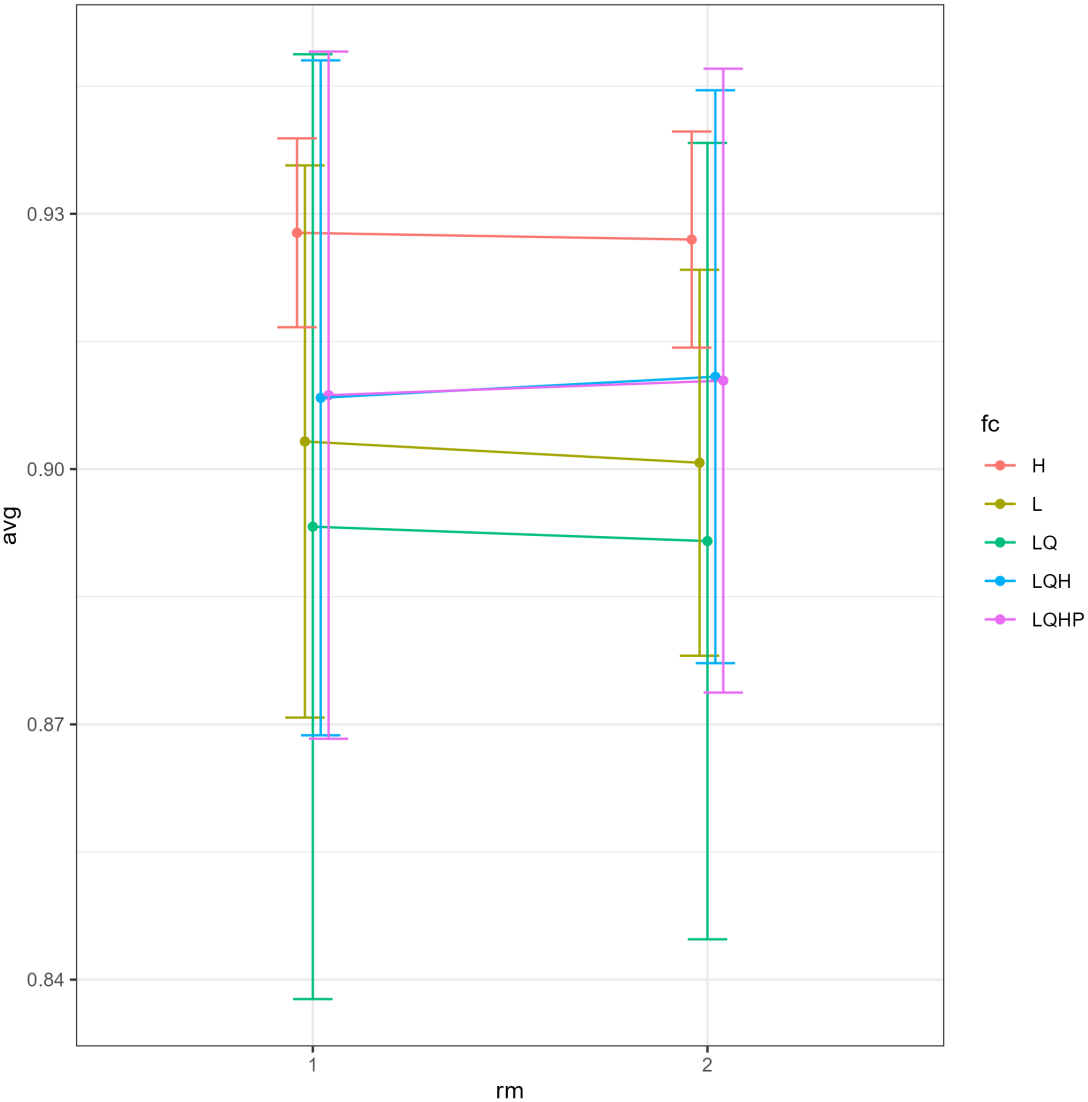
Average AUC values for *I. virgatus* in different feature classes (FC).

**FIGURE 10:**
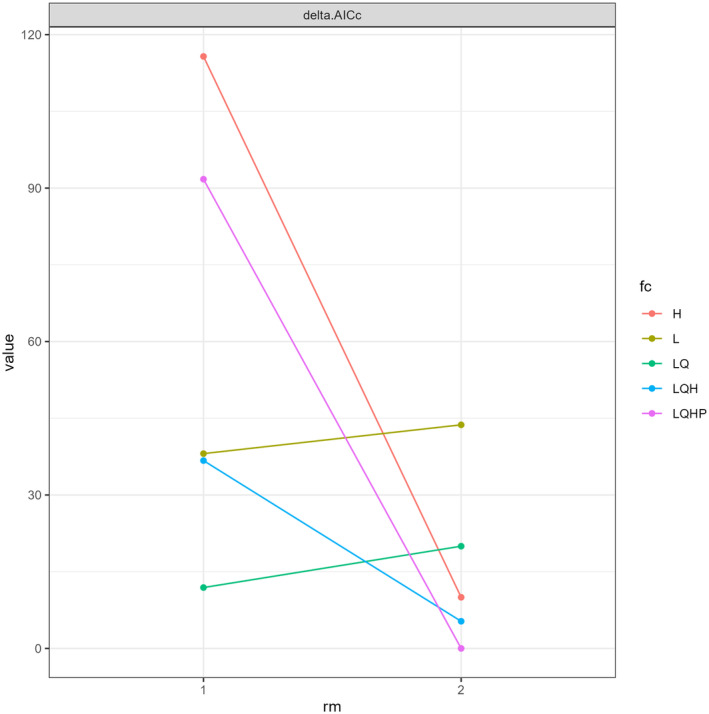
Delta AICc values for *I. virgatus*.

**FIGURE 11:**
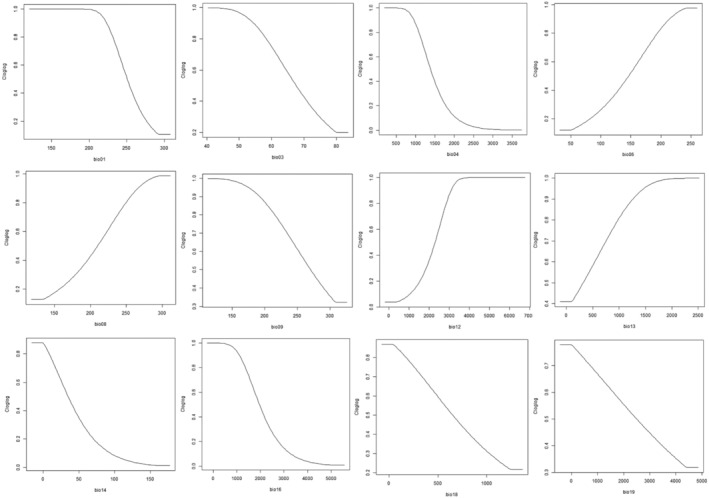
Environmental variables response curve.

The AUC values using *Wallace Ecological Modeling* ranged from 0.88 to 0.94 (Figure 9; Table 4), indicating a high level of the model's performance in predicting the distribution of *I. virgatus*. The map prediction was plotted using the combined feature class LQHP with the AUC value of 0.93. The analysis revealed delta AICc values ranging from 0 to 90 for the LQHP feature class (Figure 10). In *Wallace EcoMod*, the Annual Mean Temperature (bio1), Temperature Seasonality (bio4), Annual Precipitation (bio12), Minimum Temperature of the Coldest Month (bio6), Mean Temperature of the Wettest Quarter (bio8), and Mean Temperature of the Driest Quarter (bio9) contributed significantly to habitat suitability and distribution predictions of *I. virgatus*. In a plot of climate variables against the clog‐log (0–1) transformed model predictions, a negative linear relationship was observed, indicating that when values of the climate variables increase, the clog‐log‐transformed predictions decrease. This observation suggested that higher values of certain climate variables were associated with lower habitat suitability or probability of species occurrence (Figure 11).

**TABLE 4:** AUC values for *I. virgatus* under L, LQ, H, LQH, and LQHP feature classes using *Wallace* (current period, 1970–2000).

tune.args	fold	auc. val	auc. diff	cbi. val	or. Mtp	or.10p
fc.L_rm.1	1	0.926185	0.00481	0.9	0	0.121212
fc.L_rm.1	2	0.880301	0.065019	0.801	0.241379	0.241379
fc.LQ_rm.1	1	0.932488	0.00402	0.88	0	0.121212
fc.LQ_rm.1	2	0.853977	0.106874	0.83	0.206897	0.206897
fc.H_rm.1	1	0.935616	0.026375	0.832	0.121212	0.181818
fc.H_rm.1	2	0.919919	0.048185	0.859	0.172414	0.310345
fc.LQH_rm.1	1	0.936416	0.025268	0.886	0.151515	0.151515
fc.LQH_rm.1	2	0.880332	0.088207	0.815	0.206897	0.206897
fc.LQHP_rm.1	1	0.93724	0.025122	0.844	0.121212	0.151515
fc.LQHP_rm.1	2	0.880135	0.088155	0.829	0.206897	0.241379
fc.L_rm.2	1	0.916781	0.000776	0.95	0	0.090909
fc.L_rm.2	2	0.88471	0.050748	0.88	0.241379	0.310345
fc.LQ_rm.2	1	0.924626	0.001327	0.938	0	0.090909
fc.LQ_rm.2	2	0.858448	0.094324	0.877	0.206897	0.206897
fc.H_rm.2	1	0.935951	0.01502	0.809	0.090909	0.151515
fc.H_rm.2	2	0.917997	0.045396	0.871	0.103448	0.37931
fc.LQH_rm.2	1	0.93466	0.017527	0.834	0.121212	0.181818
fc.LQH_rm.2	2	0.887052	0.076315	0.87	0.206897	0.310345
fc.LQHP_rm.2	1	0.936317	0.015655	0.83	0.090909	0.151515
fc.LQHP_rm.2	2	0.884475	0.079265	0.897	0.206897	0.310345

### 3.5 Current Distribution Prediction Using *Wallace*

**FIGURE 12:**
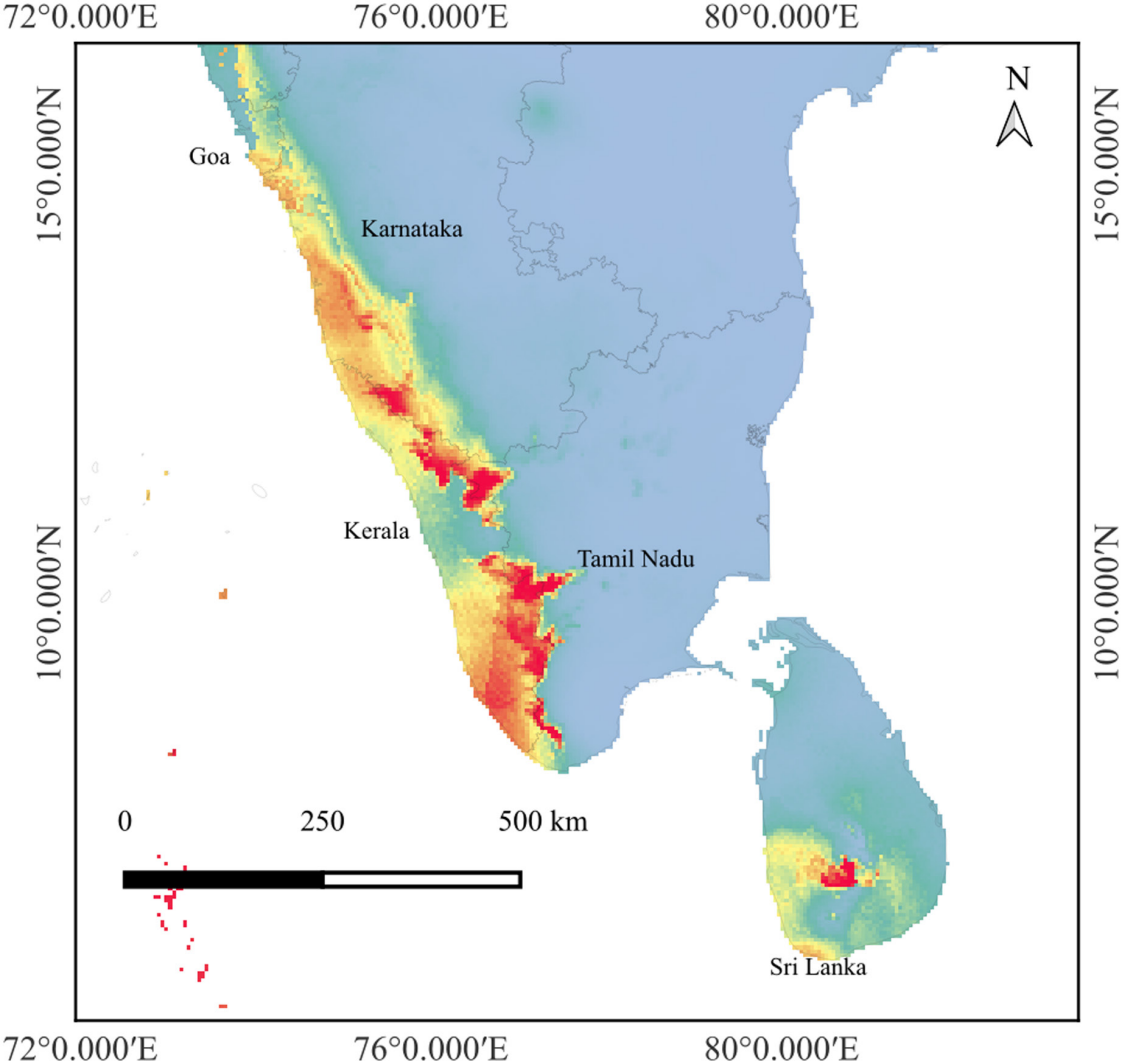
Present data (1950–2000) using *Wallace*.

In the current distribution scenario, the habitat suitability analysis revealed varying degrees of suitability for *I. virgatus* across its habitat ranges in India and Sri Lanka. In India, the HSHs totaled ~6668 km^2^, while the MSH and LSH categories covered a total of 19,821 and 89,000 km^2^, respectively (Figure 12; Table 5). The HSHs were largely centered around the SWGs and the CWGs. Similarly, in Sri Lanka, the HSHs covered ~1672 km^2^, while MSHs extended ~14,821 km^2^, predominantly within the Central Province. Based on current distribution predictions, approximately 4.76% of the WGs and about 2.55% of Sri Lanka's total area are predicted to be the HSHs for *I. virgatus*.

**TABLE 5:** Wallace Ecological modeling predicted area of suitability in India and Sri Lanka.

Province/state		RCP scenario	Highly suitable habitat (km^2^)	Moderately suitable habitat (km^2^)	Least suitable habitat (km^2^)
India	Current		6668.203	16,820.2	19,820.67
	2050	RCP26	5125.55	16,357.56	26,580.89
		RCP45	4828.12	12,331.5	14,330
		RCP60	4981.56	11,499.51	13,312.12
		RCP85	3231.2	13,283.43	12,820.11
	2070	RCP26	6232.5	17,350	24,650.9
		RCP45	4620.21	11,281.12	12,992.31
		RCP60	2928.25	12,487.32	12,324.45
		RCP85	6231.665	7881.65	9618.9
Sri Lanka	Current		1671.698	14,820.89	14,820.89
	2050	RCP26	1312.586	2350.55	16,555.23
		RCP45	3100.88	3400.1	4500.25
		RCP60	2478.21	3180.21	4671.12
		RCP85	678.81	3233.3	4730.19
	2070	RCP26	1781.83	3223.4	8300.25
		RCP45	3671.1	2530.224	3621.886
		RCP60	1224.45	2650.83	6230.09
		RCP85	2397.58	3082.54	3325.8

### 3.6 Future Distribution Prediction of *I. virgatus* Using *Wallace EcoMod*

**FIGURE 13:**
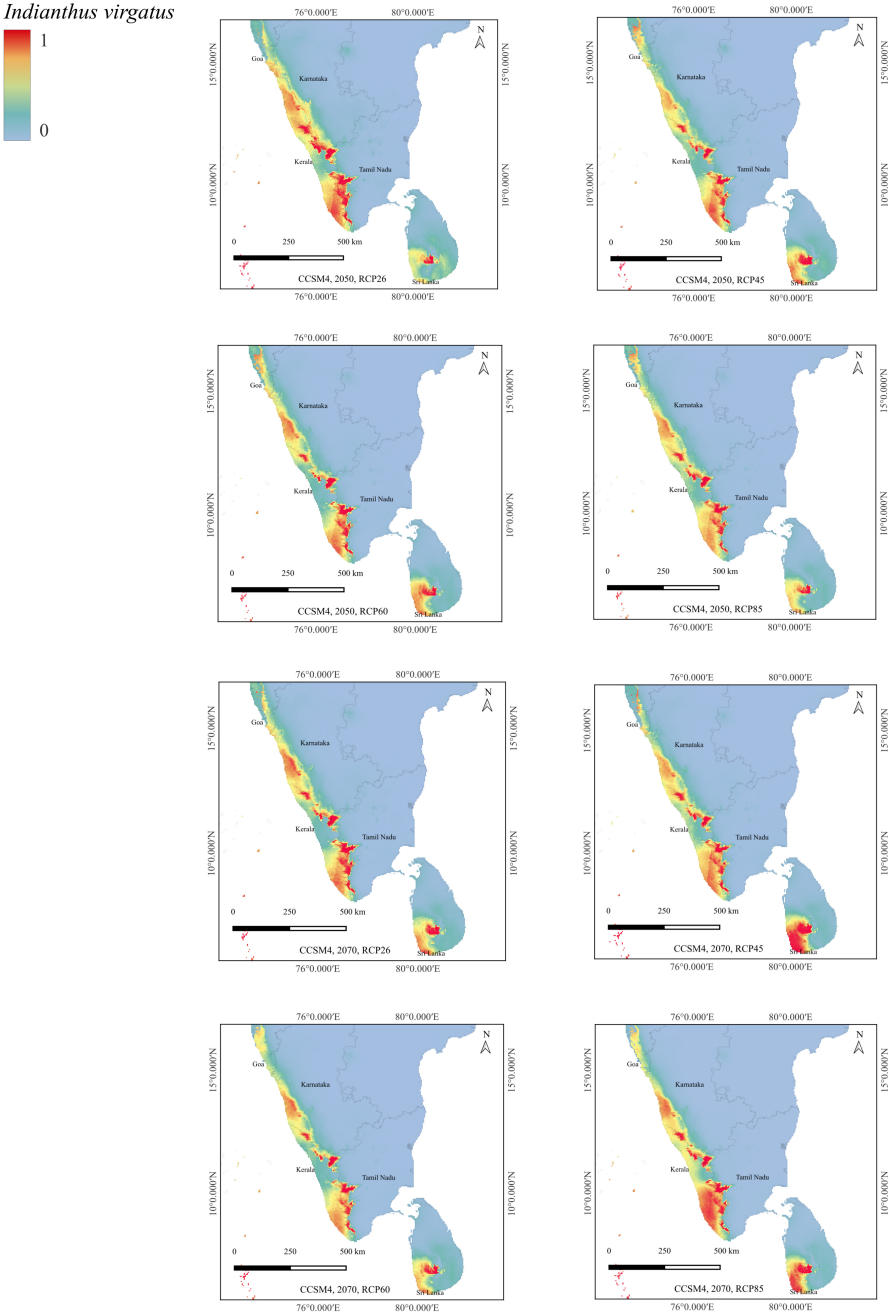
Future predictions for *I. virgatus* under CCSM4 2050 & 2070 RCP26,45,60,85 scenarios using *Wallace* modeling.

Future distribution prediction using *Wallace EcoMod* highlighted a substantial transformation in habitat suitability for *I. virgatus* across India and Sri Lanka under different RCP scenarios (Figure 12; Table 5). The model predicted a diminishing trend in the HSH availability for *I. virgatus* in India across all RCPs in 2050 and 2070, ranging from a minimum of −6.53% to a maximum reduction of −55.90%. In contrast, the HSH in Sri Lanka is predicted to be expanded under various RCPs in 2050 and 2070. Specifically, under RCP45 (2070), the model predicted a 119% increase in the HSH category. The area under MSH and LSH, however, is predicted to decline under all RCPs in 2050 and 2070. Under all RCPs in 2050 and 2070, there was a shift in the centroid of *I. virgatus* distribution from the Central Province to the Western and Southern provinces (Figure 13).

## 4 Discussion

The present study predicted the potential suitable habitats of *I. virgatus* in India and Sri Lanka for the current and future climate projections, using two different approaches, viz., *MaxEnt* and *Wallace EcoMod*. Both models exhibited higher AUC values, indicating the models' ability to predict accurately the distribution of *I. virgatus*. AUC value serves as a predictive metric for assessing model performance across various data sets, including training, testing, and random predictions. AUC values ≥ 0.90 typically indicate highly satisfactory model performance (Zhang and Wang 2023). Therefore, the predictive performance of both models used in the present study can be considered highly satisfactory. Furthermore, in *Wallace EcoMod*, the Lower delta AICc values obtained in this study highlight the model's accuracy in predicting the suitable habitat for *I. virgatus* (Yan, He et al. 2021; Yan, Wang et al. 2021; Mao et al. 2024). In model selection and evaluation, sample size and feature classes like LQHP play pivotal roles in determining AICc values (Yan, He et al. 2021; Yan, Wang et al. 2021; Li et al. 2024). In general, larger sample sizes yield more reliable parameter estimates, lowering uncertainty and often resulting in lower AICc values (Garrido et al. 2022).

### 4.1 Environmental Parameters

According to our study, the distribution of *I. virgatus* in India and Sri Lanka is influenced by a set of bioclimatic parameters, each holding significance in determining the species' habitat preferences and ecological niche. *MaxEnt* and *Wallace EcoMod* emphasized the importance of temperature and precipitation‐related variables in determining *I. virgatus* distribution. Temperature Seasonality (bio4) and precipitation in the coldest quarter appeared to be the most important variables in both models, showing the species' preference for wet and aseasonal zones that lack a defined dry season. Over the early to mid‐Miocene, much of India and Sri Lanka was covered in humid forests, which gradually diminished over the late Miocene and Pliocene, leaving wet zone species isolated in smaller regions (Meher‐Homji 1977).

*I. virgatus* may represent one of those relict species confined to the wet zones of the Western Ghats–Sri Lanka biodiversity hotspot. The complex geological history and diverse landscapes within the WGs provide greater variation in rainfall, temperature, and seasonality from south to north, west to east, and across elevation gradients. For example, wet forest formations can be found at lower elevations, and they are complemented by heavy rains with early onset and late retreat of the monsoon, resulting in reduced seasonality. At mid‐ and higher elevations, the wet zones are augmented by lower temperatures (elevation effect) and precipitation in the form of mist, fog, clouds, and occult precipitations, which further contribute to mitigating the effects of dryness.

This could explain *I. virgatus*' current distribution in the WGs, which is found across different elevations (300–1500 m) but exclusively in moist zones without a distinct dry season. The absence of *I. virgatus*, perhaps the whole Marantaceae, in the NWGs could be attributed to the extended dry season (up to 7 months) despite receiving a comparable amount of rain during the monsoon. Thus, extreme precipitation events over short durations rather than long‐term averages may be deleterious, as we found that high precipitation during specific quarters (bio16, bio18) is associated with decreased habitat suitability for *I. virgatus*. Overall, the onset and retreat of the monsoon, the length of the dry season, and elevation appear to play a major role in the wet forest formations and distribution of wet zone species like *I. virgatus* in the WGs.

### 4.2 Current Distribution

The current distribution prediction of *I. virgatus* revealed a relatively lesser proportion of the total available area as highly suitable, centered primarily around the southern part of the CWGs, where elevations vary from 500 to 1500 m (Ramachandra et al. 2018). The relatively higher annual rainfall (~4000–7000 mm) (Srinivas and Parthasarathy 2000), the absence of a distinctive dry season, and the availability of perennial water sources in this region of the WGs might offer a HSH for *I. virgatus*. The models also indicated the occurrence of LSHs in the NWGs, where the presence of Marantaceae has not yet been recorded (Gupta et al. 2024; Debnath and Vijayan 2024), indicating that the presence of *I. virgatus* may extend beyond the CWGs.

While the HSHs in the SWGs were rather scattered in the *MaxEnt* model, the *Wallace EcoMod* projected the occurrence of HSH throughout the SWG. This is to be expected as seasonality steadily rises from south to north along the WG. Furthermore, the SWG has higher elevation gradients and provides better habitats than the CWGs and NWGs (Page and Shanker 2020; Bharti et al. 2021). Similarly, the western side of the SWGs is projected to be more suitable than its eastern counterpart due to diverging patterns in rainfall and seasonality (Venkatesh et al. 2021).

For Sri Lanka, both models predicted the centroid in the Central and Sabaragamuwa Provinces, which contain virtually all of the country's rainforest (800–1500 m) (Premakantha et al. 2021; Abeyrathne et al. 2024). Since this species has already been declared Critically Endangered (CR) in Sri Lanka, the predicted suitable habitats will substantially aid in its conservation and potential reintroduction into the wild.

### 4.3 Future Distribution

Different RCPs represent different future trajectories of greenhouse gas emissions and concentrations, which in turn influence climate change outcomes. Both models predicted a considerable loss of *I. virgatus* HSHs in India and Sri Lanka under various RCP scenarios between 2050 and 2070. While the *MaxEnt* model predicted a few range expansions in the HSH category under a few RCP scenarios for India, the *Wallace* model predicted a sharp decline in HSHs across all RCP scenarios considered for 2050 and 2070, indicating the negative impact of climate change on the distribution of *I. virgatus* in India. The shift in the centroid towards the SWGs was also evident, indicating the possible shifts toward higher altitudes as a response to climate change. Earlier studies have also reported species shifting towards high‐altitudinal zones in response to changing environments (e.g., Sekercioglu et al. 2008; Ye et al. 2020; Ouyang et al. 2022; Zhang et al. 2023).

*Wallace EcoMod*, on the other hand, predicted a large range expansion in the HSH category under several RCP scenarios in Sri Lanka, with the centroid of distribution shifting between the Central and Sabaragamuwa Provinces, which are dominated by rainforests. Overall, there is convincing evidence that species are migrating to wetter environments and higher altitudes in response to climate change. While global temperature increases are expected, Elevation‐Dependent Warming (EDW) suggests systematic differences in warming rates at different elevations rather than uniformly faster warming in mountains than in lowlands (Diaz and Bradley 1997; Bradley and Diaz 2021; Qixiang, Wang, and Fan 2018; Rangwala and Miller 2012; Vuille and Bradley 2000). Changes in warming rates with elevation will also affect atmospheric static stability and precipitation patterns (Frierson et al. 2006). This phenomenon may impact the distribution of *I. virgatus*, as varying warming rates at different elevations could alter local climate conditions and, thus, habitat suitability. Since *I. virgatus* occurs at elevations ranging from 300 to 1500 m, elevation‐dependent alterations may have a considerable impact on its distribution (Toledo et al. 2021).

Nonetheless, SDM typically just depicts the fundamental niche, not the real niches. Other factors, including land‐use and land cover changes, plant–animal interactions, inter‐specific competitions, dispersal abilities, pollination guilds, and plant functional traits, play an important role (Kostikova, Salamin, and Pearman 2014; Hervías‐Parejo et al. 2020; Hederström et al. 2024). For instance, a large proportion of HSHs of *I. virgatus* in the WGs are utilized for commercial plantations like tea, coffee, and rubber. According to Pradeep, Jacob, and Annamalainathan (2020), ~5500 km^2^ of land is rubber plantation in the Kerala state, which comprises the major share of the SWGs.

The rainforests of Sri Lanka are no exception since they are vanishing at alarming rates, leaving *I. virgatus* and other wet forest species in jeopardy (Sanjeewani et al. 2024). The vast majority of the tea plantations of Sri Lanka are centered around the rainforests (Kottawa‐Arachchi and Wijeratne 2017). For instance, the SDM‐predicted suitable habitats for tea plants in Sri Lanka (Jayasinghe and Kumar 2019) closely match the HSHs of *I. virgatus*, indicating possible niche overlap. Although there is an increasingly suitable habitat predicted for the future, it would still be inaccessible for species like *I. virgatus* due to the extensive coverage of plantations in their potential niche and the species' lack of long‐distance dispersal. Plants with autochory, like *I. virgatus*, are typically more sensitive to habitat fragmentation than other plant species (Lu et al. 2012). Most of the relict plants have become extinct due to climatic fluctuations and topographic changes, and only a few taxa remain, which are confined to smaller regions (Tiffney 1985; Sang‐Joon, Hi‐I, and Byong‐Kwon 1997; Wen et al. 1997; Shen et al. 2022). The impact of climate change and ever‐increasing anthropogenic pressure will keep such species at bay unless conservation measures are implemented.

### 4.4 *MaxEnt* and *Wallace* Modeling

We observed a noticeable difference in the current and future distribution predictions of *I. virgatus* between *Wallace* and *MaxEnt* models using the same dataset. For instance, the predictions of HSHs for *I. virgatus* in India produced highly contrasting results between the two models. Besides the extent of suitable habitats, the regions marked for each habitat category also varied disproportionately. The observed variations in habitat suitability predictions between *MaxEnt* and *Wallace* can be attributed to several key factors. Primarily, differences in model assumptions regarding species‐environment relationships may lead to inconsistencies in predicted habitat suitability. *MaxEnt* and *Wallace* may employ or prioritize different environmental variables, influencing their interpretations of habitat preferences (Reuber 2023; Gerstner et al. 2024). Secondly, disparities in how geographic features are represented and analyzed can significantly impact predicted habitat areas. Variations in spatial interpolation techniques like the *spthin* (spatial partitioning) features of *Wallace* or the treatment of categorical variables may result in divergent estimations of habitat suitability (Steen et al. 2021; Valavi et al. 2023; Kass et al. 2023). Finally, differences in parameter settings, such as regularization multiplier strategies or threshold values, can exert considerable influence on the final predictions in *Wallace Ecomod* (Kass et al. 2021; Moradi, Ashrafzadeh, and Naghipour Borj 2024). Although *MaxEnt* and *Wallace* are optimized to utilize similar parameter configurations, they exhibit minor differences in reflecting varying levels of conservatism or sensitivity in their predictions.

## 5 Conclusion

The present study predicted suitable habitats of *I. virgatus* in India and Sri Lanka for the current and future scenarios. The predictions of the two models used in the study diverged greatly, especially in predicting the future distribution under various climate change regimes. These disparities in the predicted role of environmental variables can be attributed to several factors. One key consideration is the underlying algorithm and modeling approach employed by each tool. However, *Wallace EcoMod* operates within the framework of *MaxEnt*. Additionally, differences in data pre‐processing, model parameterization using different feature classes like LQHP, and the interpretation of environmental variables could also contribute to the observed differences. Nonetheless, both models indicated the possible decline of suitable habitats in India and Sri Lanka. Although a few projections of habitat expansions are predicted, those habitats might still be inaccessible, given this species' poor dispersal strategies. Further, this species niche overlaps with commercial plantation crops like tea and coffee, which may further fragment its distribution. Autochorus species like *I. virgatus* are typically more sensitive to habitat fragmentations than species with other dispersal strategies. The impact of land use dynamics, dispersal capabilities, and adaptations is beyond the scope of this study. We, however, recommend incorporating other unstudied parameters to enhance the accuracy of future predictions. Most of the relict species have gone extinct due to climate fluctuations only a few taxa like *I. virgatus* remain but are confined to smaller, isolated pockets. Therefore, these relict species, largely confined to wet zones of the WGs and Sri Lanka, is at high risk. A policy intervention is imperative, given the species' vulnerability to climate change. The predicted suitable habitats in Sri Lanka should serve as baseline information for the possible introduction of *I. virgatus* in potentially suitable habitats.

## Author Contributions

**Shreekara Bhat Vishnu:** conceptualization (equal), data curation (lead), formal analysis (equal), funding acquisition (supporting), investigation (equal), methodology (equal), project administration (equal), resources (equal), software (lead), supervision (equal), validation (equal), visualization (equal), writing – original draft (lead), writing – review and editing (supporting). **Vivek Pandi:** conceptualization (lead), data curation (supporting), formal analysis (supporting), funding acquisition (lead), investigation (equal), methodology (equal), project administration (lead), resources (equal), software (supporting), supervision (lead), validation (supporting), visualization (supporting), writing – original draft (supporting), writing – review and editing (lead). **Indrakheela Madola:** data curation (supporting), formal analysis (equal), methodology (supporting), software (equal). **Bhathiya Gopallawa:** data curation (supporting), formal analysis (supporting), methodology (supporting), software (supporting). **Gija Anna Abraham:** formal analysis (supporting), methodology (supporting), software (equal). **Rajendiran Gayathri:** data curation (equal), formal analysis (supporting), resources (supporting). **Deepthi Yakandawala:** data curation (supporting), formal analysis (supporting), investigation (supporting), supervision (supporting), validation (supporting). **Annamalai Muthusamy:** data curation (equal), supervision (supporting), writing – review and editing (supporting).

## Conflicts of Interest

The authors declare no conflicts of interest.

## Supporting information


Data S1.

## Data Availability

The data that support the findings of this study are provided as a Supporting Information.
