# Supplementary material for: Modeling the Current and Future Distribution of Indianthus virgatus (Roxb.) Suksathan & Borchs.: A Monotypic Plant Endemic to the Western Ghats‐Sri Lanka Biodiversity Hotspot
Source: Ecol Evol. 2024 Oct 26;14(10):e70489. doi: 10.1002/ece3.70489 (PMC11512157; doi:10.1002/ece3.70489)
Supplement: Supplementary file 1 — Data S1. [file ECE3-14-e70489-s001.docx]

**Supplementary data: Compilation of 105 unique location records for *Indianthus virgatus* in India and Sri Lanka**

| **Sl. No** | **Locations** | **Lat DD** | **Long DD** |
| --- | --- | --- | --- |
| 1 | Vithura, Tiruvananthapuram | 8.67534444 | 77.0852222 |
| 2 | Kothur | 8.56754167 | 77.1391667 |
| 3 | Adirapalli, Trissur | 10.2907639 | 76.5155694 |
| 4 | Parambikulam submergible area | 10.4469889 | 76.8156917 |
| 5 | Kulathupuzha | 8.90900556 | 77.059325 |
| 6 | Chethalah | 11.7496722 | 76.2476333 |
| 7 | Chadanthode | 11.8464778 | 75.8091278 |
| 8 | Chandanthode way to Mananthavady | 11.8085 | 76.0235889 |
| 9 | Maloth | 12.3754389 | 75.3703611 |
| 10 | Vandiperiar to Pamba | 9.38088611 | 76.7777972 |
| 11 | Silent valley | 9.39172778 | 76.7903167 |
| 12 | Anachal way to Vellathuval | 9.97877222 | 77.0247806 |
| 13 | E. B Campshed area, Pooyamkutty | 10.1604778 | 76.776925 |
| 14 | Muthikulum Dam area | 10.9468194 | 76.6477667 |
| 15 | Panthenthode,Malampuzha Dam | 11.0828389 | 76.4787194 |
| 16 | Gurunathan mannu | 9.2915 | 77.00635 |
| 17 | Dt., Beenachi Estate, Block A, Govt, of M.P., Sulthan Battery, Coffee plantation | 11.6627528 | 76.2247889 |
| 18 | Ponmudi Tea Plantation | 8.75781389 | 77.1153778 |
| 19 | Devikulam, Munnar top station highway | 10.1161889 | 77.1504222 |
| 20 | Kollathirumedu, Vazhachal, Trissur | 10.2859361 | 76.6707528 |
| 21 | Palode, Kerala | 8.72443333 | 77.0247806 |
| 22 | Sathram, Periyar National Park, Idukki | 9.46215556 | 77.2368472 |
| 23 | Thattekadu Bird Sanctuary, Ernakulum | 10.1298111 | 76.6870639 |
| 24 | TBGRI, Tiruvananthapuram | 8.58084167 | 76.8345111 |
| 25 | Muthikuluam, Kerala | 9.21655556 | 76.4590778 |
| 26 | Indian Cardamom Research Institute campus, Mailadumpara, Parathode, Munnar-Kumily Hwy, Mailadumpara, Kerala 685554 | 9.88870278 | 77.1555194 |
| 27 | Peppara wildlife sanctuary, Trivundrum | 8.62341389 | 77.1356861 |
| 28 | Peruvanthanam, Idukki district, Kerala | 9.55758889 | 76.9354694 |
| 29 | Mananthavady, Thalassery Baveli Road, Aaralam Forest, Kerala 670644, India | 11.8013611 | 76.0043694 |
| 30 | Thusaragiri, Kerala 673586, India | 11.4733556 | 76.0536556 |
| 31 | Thekkuthode, Pattanamthitta | 9.26568889 | 76.9700528 |
| 32 | Poppara, Idukki | 9.98026111 | 77.20315 |
| 33 | Ariankavu, Kollam | 8.97505833 | 77.1494417 |
| 34 | Peermade ghat, Idukki | 9.57826944 | 77.0150889 |
| 35 | Edaman, Travancore district | 9.00611389 | 76.9817278 |
| 36 | Nadukani Ghats, Pathanamthitta | 10.0949028 | 76.6790694 |
| 37 | Varahi falls, Varahi foests, Karnataka | 13.6937389 | 75.0191556 |
| 38 | Agumbe, Karnataka | 13.5024472 | 75.0901389 |
| 39 | Hulikal, Karnataka | 13.7292444 | 75.0187861 |
| 40 | Yedur, Hulikal | 13.7292444 | 75.0187861 |
| 41 | Sakleshpura, Near Kadagavalli Railway Station | 12.8379556 | 75.6957361 |
| 42 | Kempahole, Hassan | 12.8259667 | 75.6102472 |
| 43 | Iruppu, Kodagu, Karnataka, besides the river, laxmana theertha | 12.077125 | 76.0114889 |
| 44 | B.R.Hills (Chamarajanagar district) | 11.9988139 | 77.1395333 |
| 45 | Devarunde, Chikmagalur | 13.0224722 | 75.6303167 |
| 46 | Shiradi Ghat | 12.8235 | 75.5318222 |
| 47 | Chikmagalur | 13.4214778 | 75.7423889 |
| 48 | Uttarakannada | 14.7936917 | 74.6868861 |
| 49 | Nilgiris | 11.4916111 | 76.7336556 |
| 50 | Inchikuzhi | 10.2377139 | 76.1613917 |
| 51 | Courtallam | 8.93591111 | 77.2820778 |
| 52 | Santhi Estate, Ouchterlony Valley | 9.68391667 | 77.1211361 |
| 53 | Kammikatti- Tinnevelly | 8.71519722 | 77.7660917 |
| 54 | Nadugani | 11.4717111 | 76.4105917 |
| 55 | Kulivayal | 11.7572528 | 76.0468833 |
| 56 | Gudalur | 11.4981889 | 76.5161306 |
| 57 | Devala, Ooty | 11.4710917 | 76.3738111 |
| 58 | Kattalaimalai, Tinnevelly | 8.69103889 | 77.3662889 |
| 59 | Karaiyar to Kannikatti, Tinnevelly district | 8.65379722 | 77.3104861 |
| 60 | Sirumalai Hills, Dindigul | 10.2789917 | 77.9986417 |
| 61 | Coimbatore | 11.0035722 | 76.9362056 |
| 62 | Kandal, Ooty, Tamil Nadu | 11.4063139 | 76.6780556 |
| 63 | Valparai, Tamil Nadu | 10.3384583 | 76.9658528 |
| 64 | Dongurli, Molem National Park | 15.3778639 | 74.2297889 |
| 65 | Manamboli lower | 10.3623611 | 76.9060972 |
| 66 | top slip | 10.4690667 | 76.8401111 |
| 67 | Shendurney wildlife sanctuary | 8.85753333 | 77.2171139 |
| 68 | kerehalli | 12.9987861 | 75.8998556 |
| 69 | Siruvani Tamil Nadu | 10.9357833 | 76.6909778 |
| 70 | Kumili | 9.61253889 | 77.1787889 |
| 71 | Peredeniya | 7.26797778 | 80.0952667 |
| 72 | Attayar (TVM) | 8.62223611 | 77.2173111 |
| 73 | Attathodu (Pattanamthitta) | 9.40268611 | 77.024475 |
| 74 | Agasthyamalai | 8.88333333 | 77.1011 |
| 75 | Priya Estate (Kollam) | 8.97491944 | 77.1494917 |
| 76 | Chanapara (TVM) | 8.84334167 | 76.947175 |
| 77 | Sirendri (Silent Valley) Palakkad | 11.0775389 | 76.4223 |
| 78 | Bonallord (TVM) | 8.68041944 | 77.1673083 |
| 79 | Kulathupuzha (Kollam) | 8.90892778 | 77.0593333 |
| 80 | Mottamoodu, Near Ponmudi | 8.44993889 | 77.0254028 |
| 81 | Pamba | 9.41666667 | 77.0833333 |
| 82 | Mukkali (Palakkad) | 11.0586306 | 76.5401528 |
| 83 | Aranamudi, Sabarigiri | 9.35266389 | 76.9904528 |
| 84 | Malakkappara | 10.2783028 | 76.8583972 |
| 85 | Kulamavu, Idukki | 9.79213889 | 76.8862722 |
| 86 | Wayanad, Periya | 11.8357667 | 75.8559417 |
| 87 | 8 km from Athirumala | 8.61791667 | 77.2290222 |
| 88 | 3km from Jodupala towards Mercara (KA) | 12.4385778 | 75.6636528 |
| 89 | Pushpagiri | 12.6771111 | 75.6910278 |
| 90 | Near Tamara Coorg resort | 12.226203 | 75.647327 |
| 91 | Kithulaga | 6.997798 | 80.324571 |
| 92 | Pelmadulla | 6.661477 | 80.495497 |
| 93 | Munnar | 9.861596 | 77.164742 |
| 94 | Vandanmedu | 9.732908 | 77.147704 |
| 95 | Udumbanchola | 9.811307 | 77.150876 |
| 96 | Vagamon | 9.678098 | 76.903146 |
| 97 | Kuttikanam | 9.577206 | 76.967898 |
| 98 | Peruvathanam | 9.554298 | 76.939085 |
| 99 | Mundakayam | 9.537314 | 76.893642 |
| 100 | Thavakkal | 8.687497 | 77.056824 |
| 101 | Nedumkadam | 9.850957 | 77.148624 |
| 102 | Papmadumpara | 9.799419 | 77.159383 |
| 103 | Ayyapankoil | 9.703359 | 77.046582 |
| 104 | Kanjar | 9.817079 | 76.802875 |
| 105 | Thalakode | 10.053047 | 76.741411 |

**Online Sources of Data Collection**

| **Sl No.** | **Details of the Repository** | **Link** |
| --- | --- | --- |
| 01 | Plants of the World Online | <https://powo.science.kew.org/taxon/urn:lsid:ipni.org:names:77098027-1> |
| 02 | India Biodiversity Portal | <https://indiabiodiversity.org/species/show/243869> |
| 03 | eFlora of India | <https://efloraofindia.com/2011/11/03/schumannianthus-virgatus/> |
| 04 | E-Flora Kerala | <https://www.eflorakerala.com/> |
| 05 | BSI Pune, Western Regional Centre Online Database | <https://bsi.gov.in/regional-centres/en?rcu=134> |
| 06 | India Flora Online | <https://indiaflora-ces.iisc.ac.in/EasternGhats/herbsheet.php?id=3904&cat=4> |
| 07 | GBIF- Global Biodiversity Information Facility | <https://www.gbif.org/species/118416435> |
| 08 | HIFP- Herbarium of the French Institute of Pondicherry | <https://ifp.plantnet-project.org/list.php?searchitem=schumannianthus> |
| 09 | KFRI – Kerala Forest Research Institute Herbarium | <http://www.kfriherbarium.in/> |

10 Flowers of India <https://www.flowersofindia.net/catalog/slides/White%20Indianthus.html>
